# Single-cell RNA sequencing analysis of human chondrocytes reveals cell–cell communication alterations mediated by interactive signaling pathways in osteoarthritis

**DOI:** 10.3389/fcell.2023.1099287

**Published:** 2023-04-04

**Authors:** Xin Kang, Kailiang Zhang, Yakang Wang, Yang Zhao, Yao Lu

**Affiliations:** ^1^ Department of Orthopaedic Surgery, Honghui Hospital, Xi’an Jiaotong University, Xi'an, Shaanxi, China; ^2^ Department of Orthopedics, The 960th Hospital of the PLA Joint Logistics Support Force, Jinan, Shandong, China

**Keywords:** osteoarthritis, cell-cell communication, single-cell transcriptomic sequencing, signaling pathway, chondrocytes

## Abstract

**Objective:** Osteoarthritis (OA) is a common joint disorder characterized by degenerative articular cartilage, subchondral bone remodeling, and inflammation. Increasing evidence suggests that the substantial crosstalk between cartilage and synovium is closely related to Osteoarthritis development, but the events that cause this degeneration remain unknown. This study aimed to explore the alterations in intercellular communication involved in the pathogenesis of Osteoarthritis using bioinformatics analysis.

**Methods:** Single-cell transcriptome sequencing (scRNA-seq) profiles derived from articular cartilage tissue of patients with Osteoarthritis were downloaded from a public database. Chondrocyte heterogeneity was assessed using computational analysis, and cell type identification and clustering analysis were performed using the “FindClusters” function in the Seurat package. Intercellular communication networks, including major signaling inputs and outputs for cells, were predicted, and analyzed using CellChat.

**Results:** Seven molecularly defined chondrocytes clusters (homeostatic chondrocytes, hypertrophic chondrocyte (HTC), pre-HTC, regulatory chondrocytes, fibro-chondrocytes (FC), pre-FC, and reparative chondrocyte) with different compositions were identified in the damaged cartilage. Compared to those in the intact cartilage, the overall cell–cell communication frequency and communication strength were remarkably increased in the damaged cartilage. The cellular communication among chondrocyte subtypes mediated by signaling pathways, such as PTN, VISFATIN, SPP1, and TGF-β, was selectively altered in Osteoarthritis. Moreover, we verified that SPP1 pathway enrichment scores increased, but VISFATIN pathway enrichment scores decreased based on the bulk rna-seq datasets in Osteoarthritis.

**Conclusion:** Our results revealed alterations in cell–cell communication among OA-related chondrocyte subtypes that were mediated by specific signaling pathways, which might be a crucial underlying mechanism associated with Osteoarthritis progression.

## 1 Introduction

Worldwide, osteoarthritis (OA) is the most common degenerative joint disorder among the aging population. It frequently results in physical pain and can eventually lead to long-term disability. According to the data from the Global Burden of Disease Study, the prevalence of OA has increased from an estimated 247 million in 1990 to more than 527 million in 2019, with a particularly high prevalence in individuals aged above 60 years (Long et al., 2022). OA is a heterogeneous disease characterized by progressive degeneration of articular cartilage combined with subchondral bone remodeling and synovial membrane inflammation (Ruan et al., 2013). Aging, obesity, trauma, and mechanical loading are the main etiologic factors of OA (Chen et al., 2017). Current interventions include anti-inflammatory drugs and painkillers to relieve the symptoms, but these do not restore degraded cartilage organs or reverse disease progression (Rannou et al., 2016). Surgical joint replacement is a well-established therapy for patients with end-stage severe symptomatic OA. Despite the considerable progress in OA research, the molecular mechanisms underlying its initiation and development remain unclear.

Single-cell RNA sequencing (scRNA-seq) technology has significantly improved the detection of cell heterogeneity during disease progression and has been widely used to study cell–cell interactions. Using the scRNA-seq analysis of chondrocytes, Ji et al. identified several molecularly defined chondrocyte populations in human OA cartilage, and three novel phenotypes (fibro-chondrocytes (FC), pre-hypertrophic chondrocytes (pre-HTC), and proliferative chondrocytes) with distinct functions correlated with worse clinical outcomes in OA (Ji et al., 2019). Combining the analysis results of bulk and scRNA-seq data from OA chondrocytes, Li et al. identified a series of key genes and unraveled their expression patterns in OA progression. The potential interaction between lncRNA-CYTOR and *NRP1* might be a crucial regulatory mechanism that links arthritic knee pain and cartilage vascularization (Li et al., 2021). Synovial joint inflammation is a distinct feature of OA, and it is associated with pain severity. Based on scRNA-seq and bioinformatics analysis, a study reported that synovial tissue from pain sites exhibited a differential transcriptomic phenotype and distinct synovial fibroblast subsets at the differential stages of OA, which contributed to fibrosis, inflammation, and neuronal growth (Nanus et al., 2021). These findings highlight the importance of scRNA-seq technology in the elucidation of the molecular mechanisms and cellular functions associated with OA. However, the effects of these chondrocyte subtype-mediated cell–cell interactions on joint function and cartilage degeneration in OA remain unclear.

In this study, we further explored the online published scRNA-seq profiles of OA knee joints to gain a deeper understanding of intercellular communication during disease progression. We systematically evaluated the heterogeneity of chondrocytes and identified cell population changes in OA. In addition, we defined the alteration in cell–cell communication patterns mediated by specific signaling pathways between intact and damaged cartilages. This study elucidated the contribution of cell-specific signaling pathways to disease progression and cartilage degradation, providing novel insights into the pathophysiology of OA.

## 2 Methods

### 2.1 Transcript quantification and filtering

Single-cell transcriptome sequencing profile GSE152805 (Sebastian et al., 2021) was downloaded from the Gene Expression Omnibus (GEO, https://www.ncbi.nlm.nih.gov/geo/) database. Samples were derived from three patients with OA and the profiles were tested on a GPL20301 Illumina HiSeq 4000 platform (*Homo sapiens*). Six single-cell chondrocyte samples were selected, including three laterally intact tibial articular cartilage (GSM4626766, GSM4626767, and GSM4626768) and three tibial articular cartilage (GSM4626769, GSM4626770, and GSM4626771) samples. Meanwhile, bulk sequencing profile GSE55235 (Woetzel et al., 2014) was downloaded from GEO database. Samples were derived from ten patients with OA and ten normal patients (ND), and the profiles were tested on a GPL96 Affymetrix Human Genome U133A Array (*Homo sapiens*).

Seurat (Satija et al., 2015) (version 4.0.5, https://www.r-projec t. org/, version 4.1) in R software was used for chondrocyte scRNA-seq data processing, and a Seurat object containing all single-cell genomic data of the six samples was created. A high proportion of mitochondrial gene expression is indicative hallmark of cell stress; hence, cells with greater than 10% unique molecular identifiers were removed (Sheng et al., 2020). Cells with less than 200 gene features or greater than 6000 gene counts were removed to exclude low-quality cells, empty droplets, and doublets. Cells with greater than 60000 gene enrichment counts were filtered. Finally, 25, 245 cells were obtained for further analysis.

**FIGURE 1:**
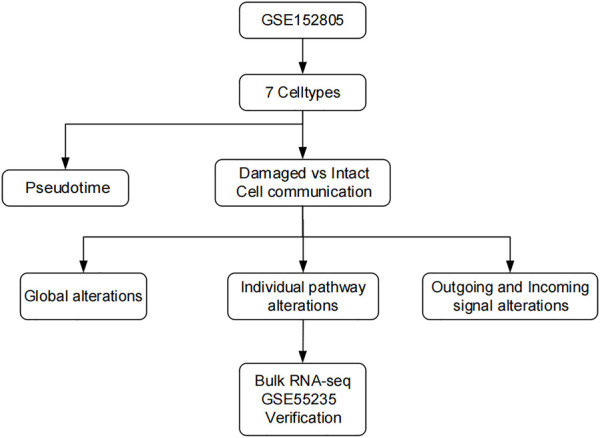
Flow chart of the study.

After data qualification, principal component analysis (Kim et al., 2018a) was performed using the most variably expressed genes from the aligned data. The Harmony (version 0.1.0) (Korsunsky et al., 2019) package was used for batch effect removal. The Seurat functions of “FindNeighbors” and “FindClusters” were used to assign cells to the optimal clusters. The cell-type clusters were visualized with t-distributed stochastic neighbor embedding, which decreased the selected principal component information to a two-dimensional data space. The flow chart for this study is shown in Figure 1.

### 2.2 Cell type annotation

The identified chondrocyte marker genes were used to manually annotate each cell cluster. The details of cell type annotation have been described in previous studies (Ji et al., 2019; Sebastian et al., 2021). Homeostatic chondrocytes (HomC) modulating cellular homeostasis were labeled with the highly expressed genes (*MMP3*, *FOSB*, and *JUN*). Hypertrophic chondrocyte (HTC) was characterized by *COL10A1*, *IBSP*, and *JUN*. The signature genes of pre-HTC were *COL10A1*, *IBSP*, *COL2A1*, and *TGF-β1*. The hallmark genes of regulatory chondrocytes (RegC) include *CHI3L1* and *CHI3L2*. The reparative chondrocytes (RepCs) were annotated with *COL2A1*, *CILP*, *COL3A1*, and *COMP*. FC were characterized by *COL1A1*, *COL1A2*, *S100A4*, *PRG4*, and *TMSB4X*. The hallmark genes of pre-fibro chondrocytes (pre-FC) include *IL11*, *COL2A1*, *CILP*, and *OGN*. The “FindAllMarkers” function was used to identify differential genes among the cell types, and the Wilcoxon rank sum test was performed for data correction.

### 2.3 Trajectory inference

The R package Monocle (version 2.22) (Qiu et al., 2017) was used to infer the trajectories of all the chondrocyte subtypes. The learning algorithm “DDRTree” implements reverse graph embedding to determine multiple decisions in an entirely unsupervised manner (Mukherjee et al., 2020). Unsupervised cell clustering and differential expression analyses were performed by Seurat et al. (Lu et al., 2020). The data were first normalized and filtered based on the minimum gene and cell-observed frequency cutoff values, and the count data were then estimated for size and dispersion. Differential gene expression analysis was performed to identify cluster genes with sex-biased expression. The most critical genes in all the clusters were selected as an input for the reverse graph embedding algorithm, and they were used to define the cell progression trajectory. The “DDRTree” was used to generate a principal tree on a single cell population, which described the global gene expression changes in cell progression and identified the branching points for determining the cell state differences.

### 2.4 Cell–cell communication analysis

The CellChat 1.0.0 package (Jin et al., 2021) in R was used to infer and quantify cellular communication involving OA by integrating single-cell expression profiles with prior knowledge of signaling ligands, receptors, and their cofactors. Ligand–receptor pairs were identified by evaluating the crosstalk probability of the ligand–receptor and perturbation tests. The cell–cell communication network was established by integrating the number of significant ligand–receptor pairs and the corresponding degree values. Changes in specific signaling pathways underlying cartilage injuries were determined by comparing the information flow of each signaling pathway between the intact cartilage and damaged cartilage tissue. The Euclidean distance was computed between any pair of shared signaling pathways.

### 2.5 Statistical analysis

All statistical analyses and data calculations were performed using the R software (Version 4.1.2). Statistical significance between two group variables was estimated using the Student’s t-test. For data that were not normally distributed, the Wilcoxon rank test was used for analysis. The similarity of any two pairs of signal pathways was calculated by Euclidean distance. *p*-values<0.05 indicated a statistically signifcant diference.

## 3 Results

### 3.1 Cell heterogeneity and trajectory reconstruction of chondrocytes

**FIGURE 2:**
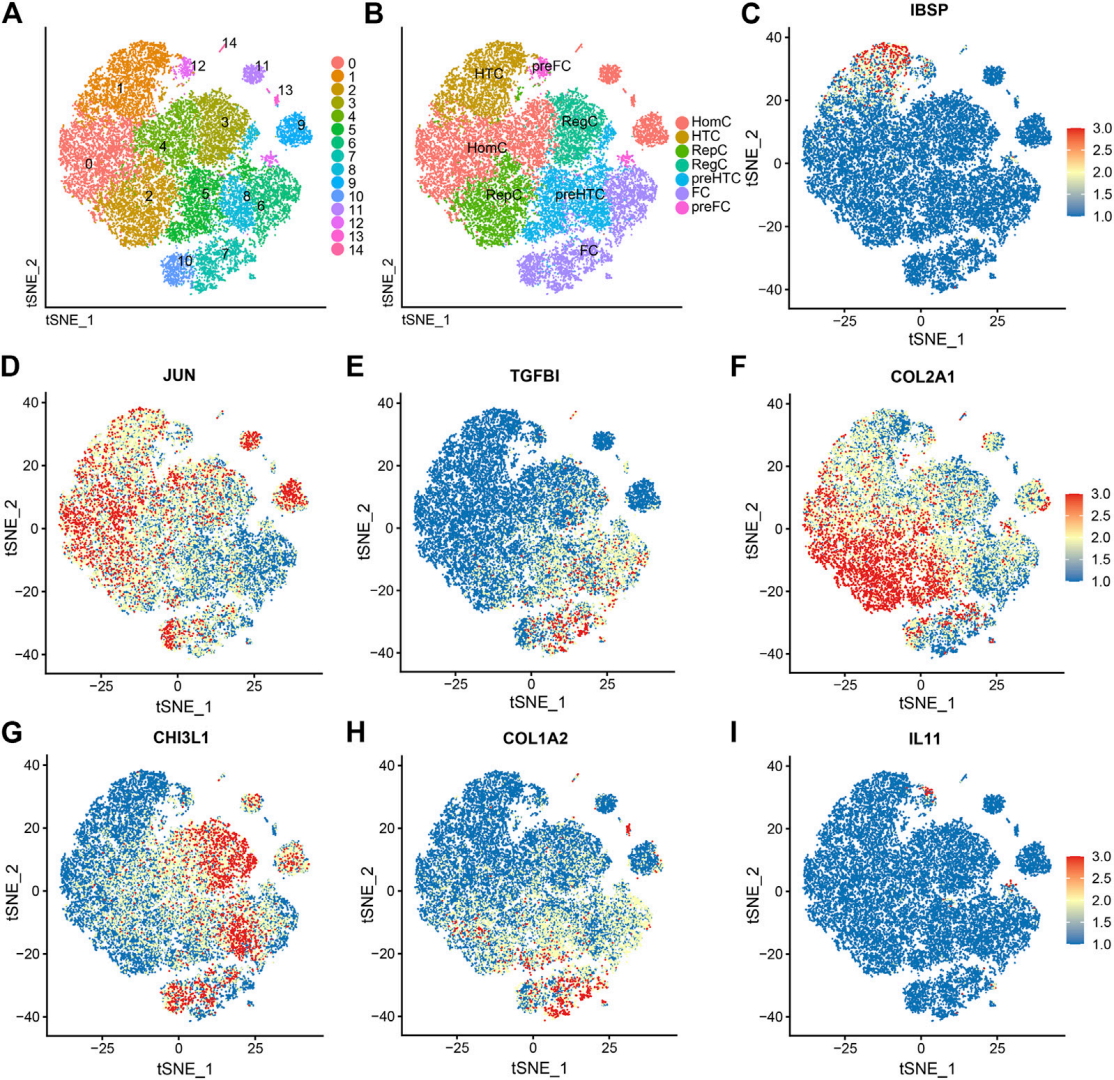
Single-cell RNA-sequencing data analysis showing the heterogeneity of chondrocytes. **(A)** T-distributed stochastic neighbor embedding (t-SNE) plots of identified chondrocyte clusters. **(B–I)** The seven subtypes of chondrocytes are colored based on the expression of well-known marker genes on the t-SNE map. The color changed from blue to red, indicating a change in gene expression from low to high.

T- SNE dimensionality reduction was used for visualization, and 25245 cells (10,927 medial damaged cells and 14,318 lateral undamaged cells) were successfully classified into 15 independent clusters (Figure 2A). The t-SNE plot of cell-type distribution in damaged and intact along with the table were showed in Supplementary Figure S2. Based on the identified marker genes, 15 chondrocyte clusters were annotated into seven subtypes: HTC (3458, 13.70%), HomC (7659, 30.34%), pre-HTC (3719, 14.73%), RepC (3342, 13.24%), RegC (2444, 9.68%), FC (4184, 16.57%), and pre-FC (439, 1.74%), and the corresponding marker gene expression of the seven cell types was *IBSP*, *JUN*, *TGF-ΒI*, *COL2A1*, *CHI3L1*, *COL1A2*, and *IL11*, respectively (Figures 2B–I).

### 3.2 Pseudo-temporal analysis

**FIGURE 3:**
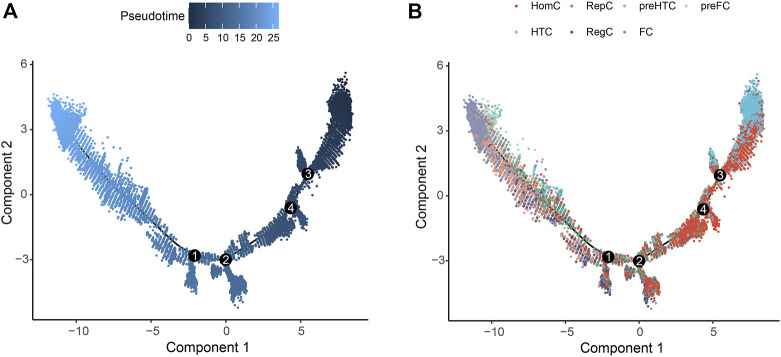
Pseudo-temporal trajectory revealing the chondrocyte progression. **(A)** Pseudo-time progression diagram with color from dark to light indicating the pseudo-time order. **(B)** Pseudo-temporal map of chondrocytes colored according to cell subtype. The horizontal and vertical axes represent two principal components. Each dot indicates a single cell, and the digit in the circle represents the predicted nodes of the different cell states in the trajectory.

Pseudo-temporal analysis was performed for the seven chondrocyte subtypes to construct the trajectory map. HomC and HTC were distributed at the beginning of the trajectory. Pre-HTC, RegC, RepC, and pre-FC existed along the trajectory, whereas FC was mainly distributed at the end (Figure 3).

### 3.3 Global alterations in intercellular signaling networks in OA

**FIGURE 4:**
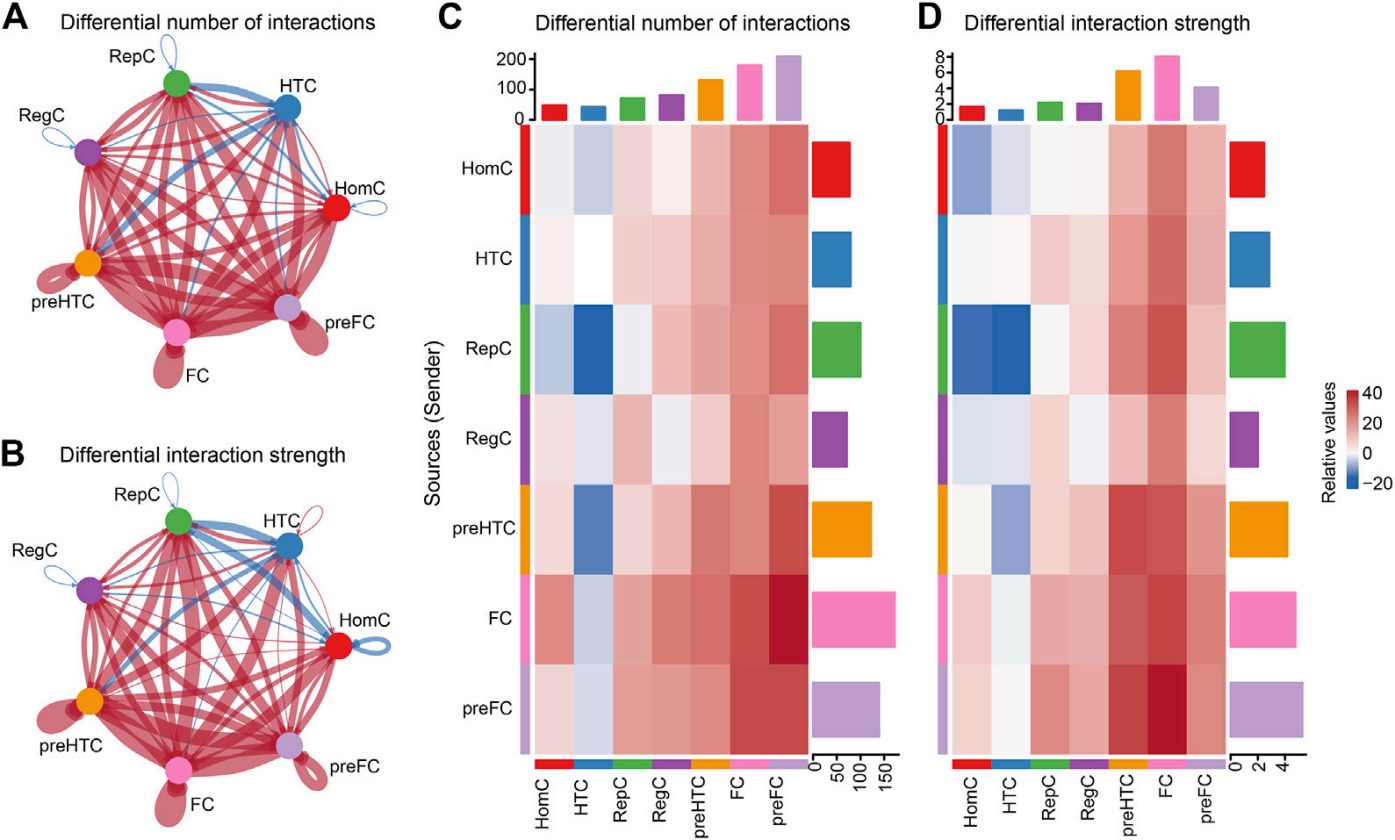
Inferences of cell–cell communication by CellChat revealing the global signaling changes between the intact and damaged cartilage samples. **(A–B)** Circular plots of the cellular interaction number **(A)** and interaction strength **(B)** between each chondrocyte subtype. The blue line indicates a decreased communication number in the damaged cartilage compared to that in the intact cartilage, while the red line indicates an increased communication number in the damaged cartilage. **(C–D)** Heat maps of the cellular interaction number **(C)** and interaction strength **(D)** between chondrocyte subtypes. The vertical axis represents the signal sender and the horizontal axis represents the signal receiver. The blue column indicates decreased communication in the damaged cartilage compared to that in the intact cartilage, while red indicates increased communication. The upper and right columns show the relative values of the interaction number and strength, respectively.

To predict OA-related pathological changes in cellular communication, we compared cellular signaling flux patterns between the intact and damaged cartilage samples. The global alteration in cellular communication between each chondrocyte subtype was quantified and visualized using CellChat. The results showed that the cell–cell interaction number and interaction strength increased in the damaged cartilage compared to those in the intact cartilage (Figures 4A,B). In contrast, the overall number of interactions between HTC and the other cell subtypes (such as HomC, pre-FC, FC, pre-HTC, and RegC) remarkably decreased in OA compared to that in the control group. Moreover, the signals sent by RepC to HomC were reduced in the damaged cartilage (Figures 4C,D).

### 3.4 Alterations in individual signaling pathways in the intact and damaged cartilage groups

**FIGURE 5:**
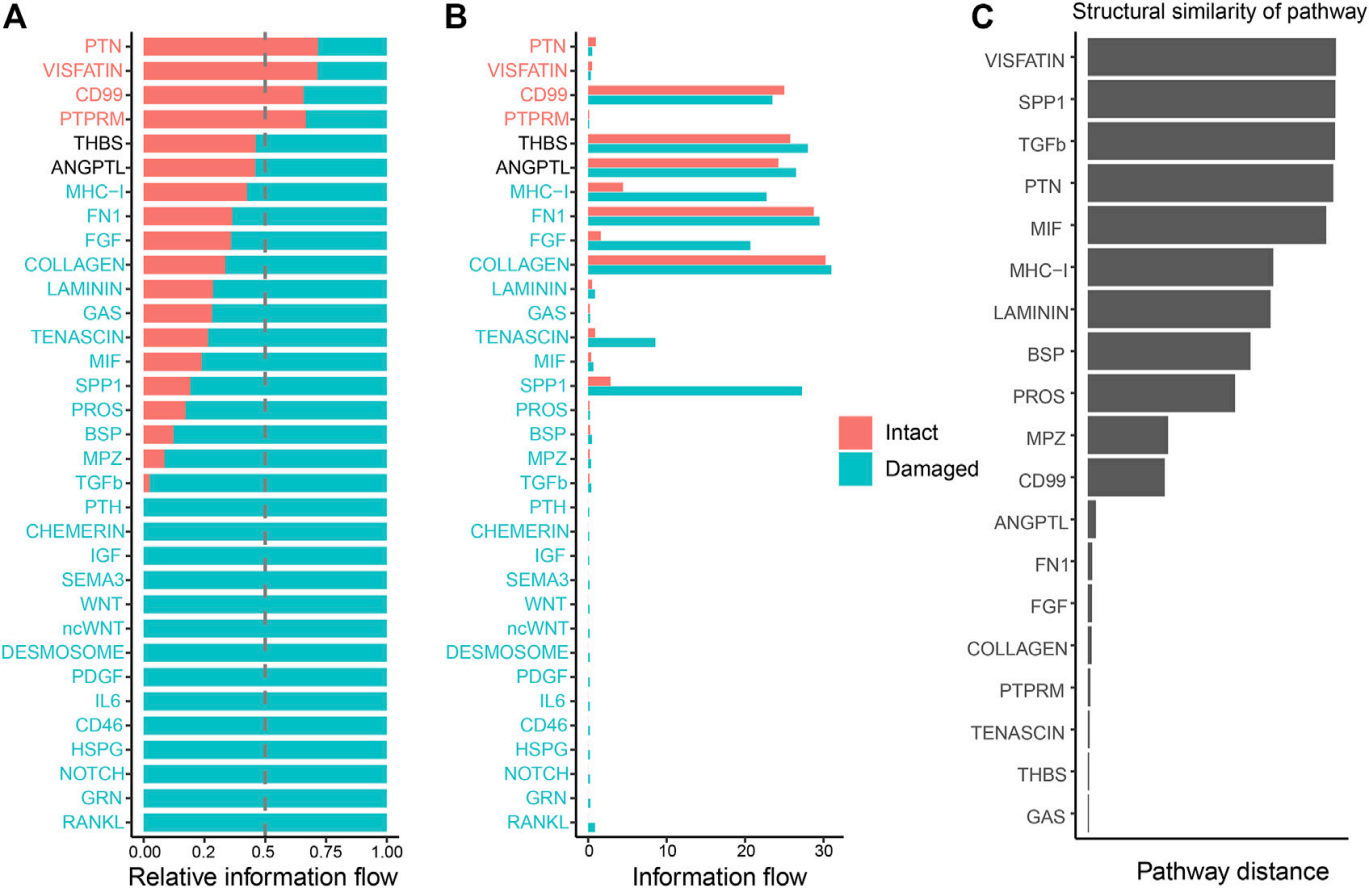
Cellular communication changes mediated by signaling pathways in osteoarthritis between the intact and damaged cartilage groups. **(A)** Bar chart showing the difference in the relative ratio of information flow in the interaction network between the intact and damaged cartilage samples. **(B)** Information flow numbers between the intact and damaged cartilage samples. The top signaling pathways, marked with red color at the top, are enriched in the intact cartilage samples, while green color-labeled signaling pathways are enriched in the damaged cartilage samples. The middle pathways marked in black are equally enriched in the two groups. **(C)** Degree of signaling pathway differences between intact and damaged cartilage samples by pairwise Euclidean distance. The Wilcoxon test was used to determine whether a significant difference between the two groups. *p*-values<0.05 indicated a statistically signifcant diference.

To detect global alterations in certain signaling pathways in OA, we calculated the information flow in each signaling pathway, which was reported as the potential communication probability of cell subgroups in the communication network (Jin et al., 2021). Compared to those in the intact cartilage, several pathways, such as PTH, CHEMERIN, IGF, SEMA3, WNT, ncWNT, DESMOSOME, PDGF, IL6, CD46, HSPG, NOTCH, GRN, and RANKL, were turned on in the damaged cartilage (Figure 5A). The activities of most pathways, such as MHC-I, FN1, FGF, COLLAGEN, LAMININ, GAS, TENASCIN, MIF, SPP1, PROS, BSP, MPZ, and TGF-β, increased in the damaged cartilage, whereas those of the other pathways, such as PTN, VISFATIN, CD99, and PTPRM, decreased. There were no significant differences in the activities of THBS and ANGPTL pathways between the two groups (Figure 5B).

The changes in the overlapping signaling pathways were also measured by computing the Euclidean distances. VISFATIN, SPP1, TGF-β, and PTN signaling pathways had values larger than those of the other pathways, indicating that they might be crucial factors in cartilage injury (Figure 5C).

### 3.5 Signaling flow patterns in each chondrocyte subtype

**FIGURE 6:**
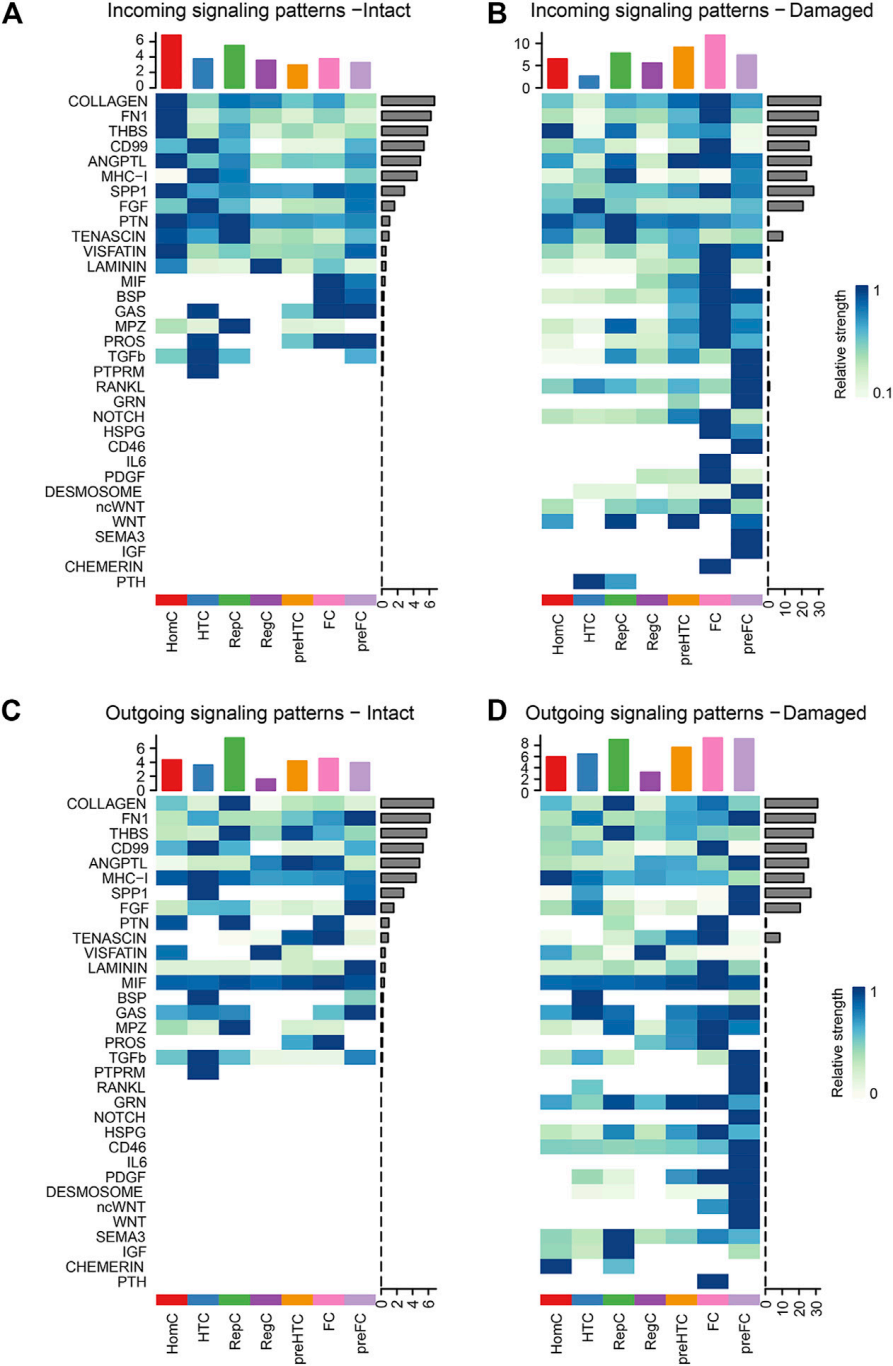
Heatmaps of the incoming **(A–B)** and outgoing **(C–D)** signaling pattern in each cell subtype mediated by individual signaling axes between the intact and damaged cartilage samples. Cell types are distributing on the horizontal axis and signaling pathway on the vertical axis. The upper and right columns are the relative strength of the vertical and horizontal axes, respectively.

To identify the signaling flow pattern in each chondrocyte subtype, the changes in outgoing and incoming signaling patterns in the intact and damaged cartilages were compared. COLLAGEN and FN1 signaling were mainly introduced into HomC in the intact cartilage and fluxed into FC in the damaged cartilage. The activities of THBS signaling pathways to pre-HTC and FC were increased in the damaged cartilage. CD99 was mainly afferent to HTC in the intact cartilage and afferent to the FC subtype in the damaged cartilage (Figures 6A,B). Moreover, the COLLAGEN signaling pathway outgoing from pre-HTC and FC was upregulated in the damaged cartilage. The efferent pattern of THBS signaling from the pre-HTC and FC decreased. The outgoing signaling pattern of CD99 decreased in HTC, and the outgoing pattern was enhanced in the FC subtype (Figures 6C,D).

### 3.6 Alterations in specific signaling pathways between the intact and damaged cartilages

According to the previous steps, we found 19 overlapping signaling pathways between the two groups: VISFATIN, SPP1, TGF-β, PTN, MIF, MHC-I, LAMININ, BSP, PROS, MPZ, CD99, ANGPTL, FN1, FGF, COLLAGEN, PTPRM, TENASCIN, THBS, and GAS. Compared to those in the intact cartilage, intercellular signaling networks were notably altered on a global scale in the damaged cartilages.

**FIGURE 7:**
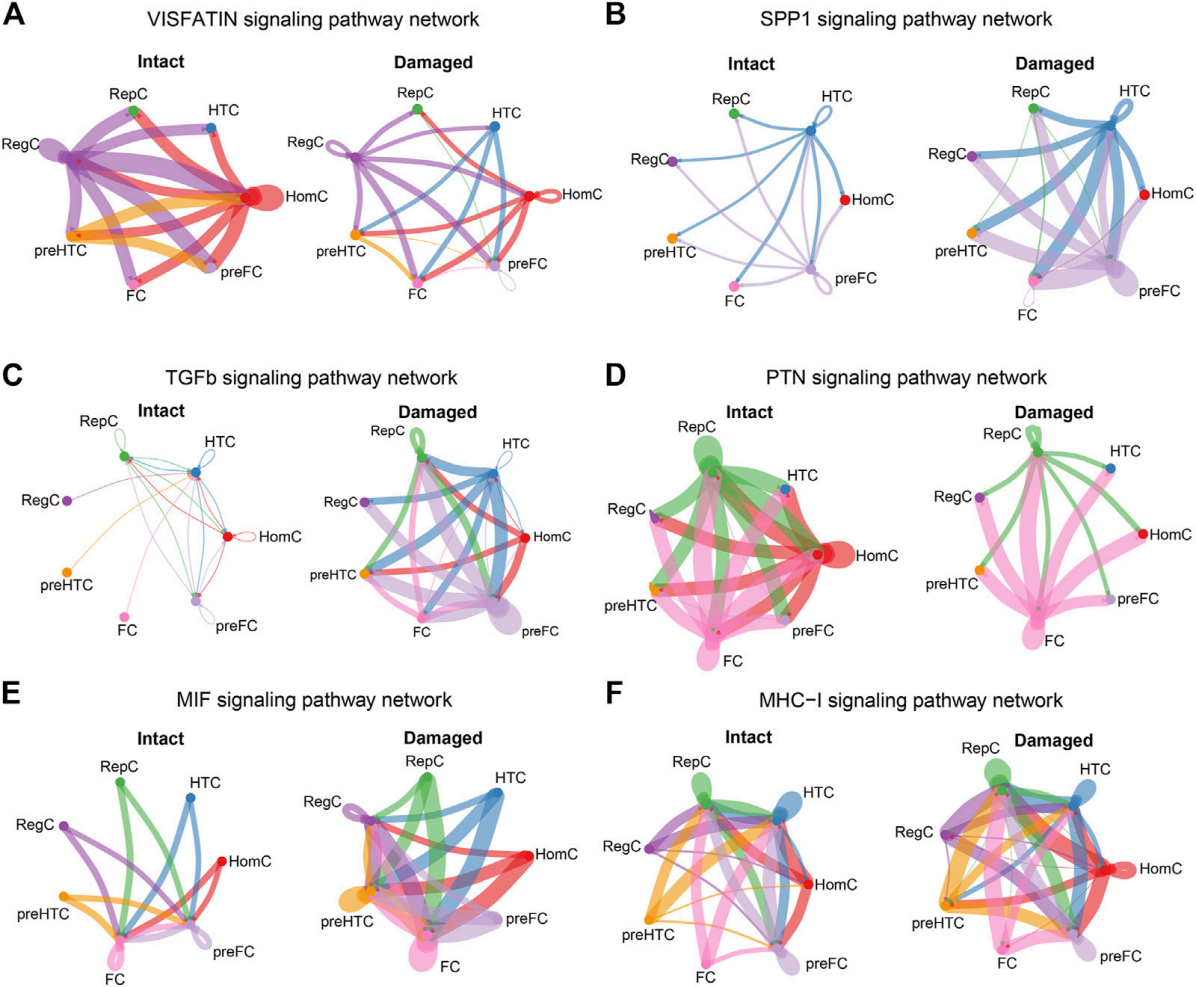
Cellular communications among the chondrocyte subtypes are significantly altered in the damaged cartilage compared to those in the intact cartilage samples. The circle plots show the obvious changes in cell communication mediated by VISFATIN **(A)**, SPP1 **(B)**, TGF-β **(C)**, PTN **(D)**, MIF **(E)**, and MHC-I **(F)** signaling pathways. Colored dots represent different cells subtype. The thickness of the lines represents the strength of cell connection, thicker the line the stronger the interaction.

Under normal conditions, RegC and HomC acted as VISFATIN signaling centers by communicating with the other chondrocyte subtypes, whereas this signal was remarkably reduced in the injured cartilage (Figure 7A). Moreover, the SPP1 signal level was enhanced in the damaged cartilage with HTC and pre-FC as signal communication centers (Figure 7B). Furthermore, the TGF-β signal mainly originated from RepC, HTC, HomC, and pre-FC in the intact cartilage. Notably, the overall abundance of TGF-β signals was significantly increased in OA and the degree of involvement of RegC, pre-HTC, and FC was enhanced (Figure 7C).

HomC was the main source of PTN signals in the intact cartilage, and the signal flux was reduced in the damaged cartilage (Figure 7D). Another group of MIF signals was visibly enhanced in the damaged cartilage, and in particular, each chondrocyte subtype increased cellular communication with RegC (Figure 7E). Moreover, the MHC-I signal level in the damaged cartilage was significantly increased, and the involvement of each cell subtype was also enhanced (Figure 7F). The circle plots for the remaining 13 overlapping signaling pathways (LAMININ, BSP, PROS, MPZ, CD99, ANGPTL, FN1, FGF, COLLAGEN, PTPRM, TENASCIN, THBS, and GAS) were displayed in the Supplementary data (Supplementary Figure S1 and Suplementary Figure S2).

### 3.7 Validation was performed in the bulk rna-seq datasets

**FIGURE 8:**
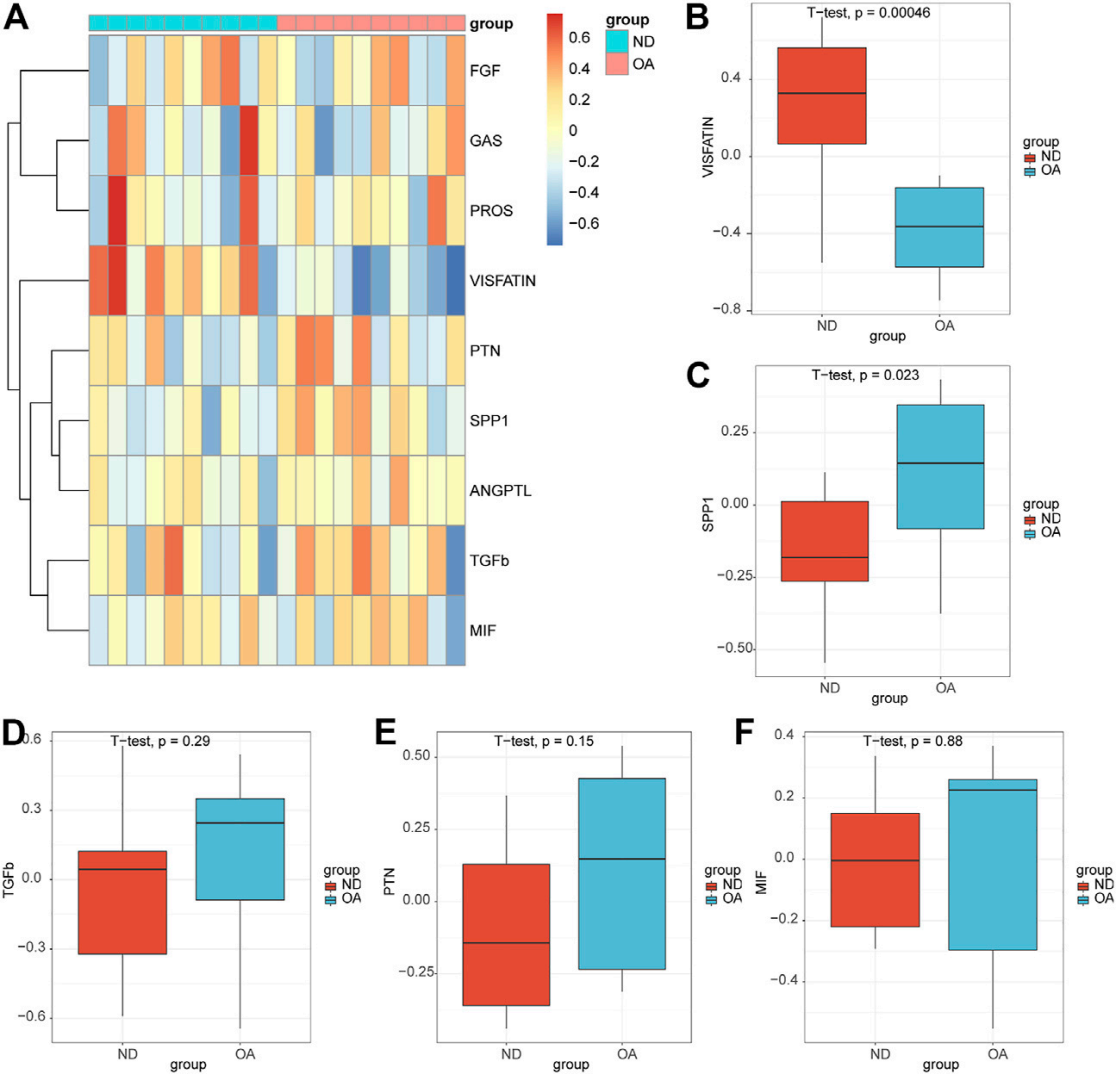
Validation of the pathways in the bulk rna-seq datasets. **(A)** Heatmap of the GSVA enrichment scores of the signaling pathways. Box plots of the enrichment scores of the VISFATIN **(B)**, SPP1**(C)**, TGFb **(D)**, PTN **(E)**, MIF **(F)** signaling pathways. The *t*-test was used to compare the diferences between two groups. *p*-values<0.05 indicated a statistically signifcant diference.

We performed GSVA enrichment analysis on OA samples and ND samples in datasets GSE55235 based on pathway information in intercellular communication, and nine signaling pathways (VISFATIN, SPP1, TGFb, PTN, MIF, PROS, ANGPTL, FGF, GAS) were enriched (Figure 8A). The box plots showed that the VISFATIN pathway (Figure 8B) enrichment scores were significantly lower (*p* < 0.05), where as the SPP1 pathway (Figure 8C) enrichment scores were significantly higher (*p* < 0.05), in the OA samples compared with those in ND samples. There were no significant differences in TGFb (Figure 8D), PTN (Figure 8E) and MIF (Figure 8F) pathways in the OA samples compared with those in ND samples.

## 4 Discussion

Progressive cartilage degeneration is a key hallmark of osteoarthritis pathogenesis, and molecular changes in articular chondrocytes have been implicated as predisposing factors for cartilage loss (Chou et al., 2020; Sebastian et al., 2021). These findings provide new insights into cellular and transcriptional alterations, providing a theoretical foundation for the development of new therapies. First, we characterized cellular heterogeneity in the scRNA-seq datasets of human osteoarthritic cartilage tissue. Using CellChat technology, we found that intercellular communication frequency and strength were globally enhanced in the damaged cartilage compared to those in the intact tissue. A series of signals were altered in OA, and based on the Euclidean distance evaluation, four signaling pathways (VISFATIN, SPP1, TGF-β, and PTN) showed the most significant changes. Additionally, the VISFATIN pathway enrichment scores were significantly lower, where as the SPP1 pathway enrichment scores were significantly higher, in the OA samples compared with those in ND samples in the bulk rna-seq datasets.

Among these altered signaling pathways, VISFATIN exhibits proinflammatory and pro-degradative effects on cartilage, and serum VISFATIN levels are increased in patients with knee OA (Laiguillon et al., 2014; Liao et al., 2016). VISFATIN increased the generation of inflammatory cytokines (such as IL-6, TNF-α, and MMPs) in human OA cartilage chondrocytes, and intra-articular injection of VISFATIN induced cartilage destruction in a mouse model (Yang et al., 2015; Philp et al., 2021). Moreover, VISFATIN can influence intracellular mechanics and dynamic catabolism in primary chondrocytes through p38 signaling-mediated GSK3β inactivation (Chang et al., 2021). However, we found that VISFATIN signaling in damaged cartilage was lower than that in intact tissue. The reduction in VISFATIN signals in chondrocytes might imply that patients had different OA stages; hence, further investigation is required.

Moreover, we found that the SPP1/OPN signal was remarkably enhanced under OA conditions, and the HTC and pre-FC seemed to be the major signal transduction players. SPP1 or osteopontin (OPN) is a non-collagen, highly phosphorylated, and glycosylated protein secreted by various cells, such as lymphocytes, chondrocytes, and synoviocytes. It is extensively expressed in the extracellular matrix of mineralized tissues, inflammatory sites, and bone and cartilage tissues. OPN was identified in severe, moderate, minor, and normal tissues (Li et al., 2016). And, there was statistical differences in the intercomparisons of OPN expression levels. In articular cartilage, the expressions of OPN was associated with different OA stages (Li et al., 2016). The levels of OPN in the serum and joint synovial fluid were notably increased in patients with OA, and increased OPN level was associated with disease severity (Gao et al., 2010; Min et al., 2021). OPN has a significant effect on joint cartilage destruction by promoting synovial angiogenesis and inducing chondrocyte apoptosis (Yumoto et al., 2002). Based on these results, upregulated OPN expression in subchondral bone could promote bone turnover and remodeling to accelerate the progression of OA, and the blockage of PI3K/AKT signaling could inhibit OPN-mediated cartilage degeneration (Lin et al., 2022). However, another study reported that OPN might have a protective effect on human OA and could inhibit OA progression by activating intracellular PI3K signaling, thereby suppressing chondrocyte apoptosis and reducing cartilage matrix component loss (Liu et al., 2020). Furthermore, OPN deficiency accelerates the severity of OA lesions, which is associated with enhanced chondrocyte senescence and apoptosis, and upregulated OA-related molecules (including proinflammatory cytokines of TNF-α, IL-1β, and several matrix-degrading enzymes) (Tian et al., 2020). These findings suggest that OPN plays a dual role in the progression of OA. Thus, one hypothesis is that elevated OPN expression may serve as an adaptive response to protect cells from the catabolic and inflammatory environment, and further progression of the degenerative process could lead to the failure of this defensive response (Sun et al., 2020). In contrast, PI3K/AKT/mTOR, a major downstream signal of OPN, has a complex role in OA (Liang et al., 2018). It is essential for the maintenance of joint function and is commonly activated during OA initiation and progression (Sun et al., 2020). It has been reported that activated PI3K signaling displayed an anti-arthritic role by promoting chondrocyte proliferation and reducing apoptosis (Huang et al., 2011; Yao et al., 2019). In contrast, the inhibition of PI3K signaling attenuates OA-related joint damage by enhancing autophagy and suppressing inflammatory responses (Li et al., 2016; Lin et al., 2018). Further studies should investigate whether OPN plays a positive or negative role in OA.

The level of another intercellular signal PTN decreased in OA-related HomC. PTN is a heparin-binding growth factor, with potent mitogenic and angiogenic activity, and a crucial regulator of cancer metastasis, bone development, and bone repair (Liedert et al., 2014). Previous studies have reported that PTN levels are increased in the serum, chondrocytes, and subchondral bone of patients with OA, indicating that PTN might be a crucial factor during disease progression (Kaspiris et al., 2013; Fadda et al., 2018). PTN also promotes endothelial cell proliferation and migration and induces angiogenesis by interacting with two main receptors, PTPRZ and αVβ3 (Fukada et al., 2006; Wang, 2020). Angiogenesis may indirectly accelerate OA by increasing inflammatory cell infiltration (MacDonald et al., 2018); hence, PTN can aggravate OA development by promoting angiogenesis. PTN expression is also elevated after bone damage, and in cases of fracture healing, the upregulated PTN expression is responsible for the recovery of the injured area through the promotion of local angiogenesis (Peterson et al., 2004; Wang, 2020). Moreover, PTN-induced human bone marrow mesenchymal stromal cells differentiate into a hypertrophic state during chondrogenesis, which reinforces bone regeneration (Bouderlique et al., 2014). Indeed, PTN expression in chondrocytes is increased in early OA stages, whereas it is lower in later stages (Pufe et al., 2003), and they reported that PTN might be involved in cartilage repair in earlier OA stages. Importantly, the expression of PTN in the early stages induced chondrocyte proliferation and clustering, leading to increased collagen synthesis and bone formation, whereas cartilage degeneration considerably exceeded the cartilage repair effect at later stages (Pufe et al., 2007). Taken together, we suggest that the reduction of PTN levels in HomC might be linked to attenuated bone repair roles in OA progression.

Moreover, we found that TGF-β signaling in the chondrocytes was notably increased, and the frequency of signal flux in the damaged cartilage was enhanced among RegC, pre-HTC, and FC. TGF-β is a pleiotropic cytokine, and its signaling is a crucial regulator of most biological processes in mammals. HTCs contribute to the development of OA (Thielen et al., 2022). Compressive loading-induced TGF-β signaling is a default mode and self-regulatory system that prevents articular chondrocyte hypertrophic differentiation and cartilage destruction (van der Kraan, 2017). Mechanistically, the binding of active TGF-β and chondrocyte receptor activin receptor-like kinase 5 (ALK5) stimulates the expression of latent TGF-β1 and ALK5, while ALK1 levels are downregulated (Finnson et al., 2008). ALK5 signaling blocks chondrocyte hypertrophy through SMAD2/3 signaling, whereas ALK1 stimulates chondrocyte hypertrophy through SMAD1/5/8 (Ferguson et al., 2000; Li et al., 2006; Dexheimer et al., 2016). Age-related alterations in the ALK5, ALK1 ratio, and loss of loading-induced TGF-β signaling could facilitate cartilage degeneration and increase the susceptibility of cartilage to OA development (Blaney Davidson et al., 2009; Tominaga and Suzuki, 2019). Moreover, a relatively high concentration of TGF-β in the joints leads to osteophyte formation and synovial fibrosis. Systemic TGF-β inhibition could result in progressive inflammatory effects; hence, joint-specific inhibition was considered as an alternative therapy for OA. For example, TissueGene-C, a cellular therapeutic method for OA, in which retrovirally transduced allogeneic chondrocytes are used to overexpress TGF-β1, displayed the capability to induce cartilage repair in an animal model; however, improvements in structural outcomes were limited in phase III clinical trials (Kim et al., 2018b). Nanoengineered mesenchymal stem cells transfected with a plasmid encoding TGF-β1 significantly facilitated cartilage repair, indicating a potential strategy to overcome the current limitations associated with OA treatment (Cai et al., 2022). However, using the bulk sequencing approach, we could not confirm a significant difference between OA and non-OA patients. The reason for this result may be due to the small sample size.

This study has some limitations. For example, due to space limitation, only some of the highly relevant signaling pathways were selected for analysis. Other pathways that have significant alterations in OA will be taken into account in future studies (Supplementary Figure S1, S2). Furthermore, owing to the difficulties in obtaining non-OA samples and because the intact cartilage from OA might display an inflammatory status, we could not directly evaluate the differences in cell patterns between the diseased and healthy states. In addition, further experimental verifications are necessary to elucidate the biological functions of these predicted altered signaling pathways in damaged cartilage. Moreover, we would need to perform more careful examinations of altered signaling pathways in damaged cartilage by combining *in vitro* and *in vivo* techniques (such as quantitative real-time PCR, western blot, immunofluorescence and immunohistochemistry assays).

In summary, the results of this study highlight the effect of signaling pathways changes in damaged and intact cartilage in OA. Many pathways have been explored, and we verified that SPP1 pathway enrichment scores increased, but VISFATIN pathway enrichment scores decreased based on the bulk rna-seq datasets in OA. Moreover, the inhibitors of some of these pathways are already in clinical trials (Robbins et al., 2021). The elucidation of OA-related cellular communication might provide a guide for the development of therapeutic strategies for existing diseases. Further studies are needed to obtain reliable conclusions and identify the exact mechanism of cell–cell communications in chondrocyte populations in cartilage degradation in OA.

## Data Availability

The datasets presented in this study can be found in online repositories. The names of the repository/repositories and accession number(s) can be found in the article/Supplementary Material.

## Abbreviations

ALK5, activin receptor-like kinase 5; FC, fibro-chondrocytes; GEO, Gene Expression Omnibus; HomC, homeostatic chondrocyte; HTC, Hypertrophic chondrocyte; OA, osteoarthritis; pre-HTC, pre-hypertrophic chondrocytes; RepC, reparative chondrocytes; scRNA-seq, single-cell transcriptome sequencing; RegC, regulatory chondrocytes; pre-FC, pre-fibro chondrocytes.

## References

[B1] Blaney Davidson E. N. Remst D. F. G. Vitters E. L. van Beuningen H. M. Blom A. B. Goumans M. J. (2009). Increase in ALK1/ALK5 ratio as a cause for elevated MMP-13 expression in osteoarthritis in humans and mice. J. Immunol. 182 (12), 7937–7945. 10.4049/jimmunol.0803991 19494318

[B2] Bouderlique T. Henault E. Lebouvier A. Frescaline G. Bierling P. Rouard H. (2014). Pleiotrophin commits human bone marrow mesenchymal stromal cells towards hypertrophy during chondrogenesis. PLoS One 9 (2), e88287. 10.1371/journal.pone.0088287 24516627 PMC3917886

[B3] Cai Y. Wu C. Ou Q. Zeng M. Xue S. Chen J. (2022). Enhanced osteoarthritis therapy by nanoengineered mesenchymal stem cells using biomimetic CuS nanoparticles loaded with plasmid DNA encoding TGF-β1. Bioact. Mater 19, 444–457. 10.1016/j.bioactmat.2022.04.021 35574050 PMC9079106

[B4] Chang S. F. Huang K. C. Lee K. H. Chiang Y. C. Lee W. R. Hsieh R. Z. (2021). Effects of visfatin on intracellular mechanics and catabolism in human primary chondrocytes through glycogen synthase kinase 3β inactivation. Int. J. Mol. Sci. 22 (15), 8107. 10.3390/ijms22158107 34360874 PMC8348639

[B5] Chen D. Shen J. Zhao W. Wang T. Han L. Hamilton J. L. (2017). Osteoarthritis: Toward a comprehensive understanding of pathological mechanism. Bone Res. 5 (16044), 16044. 10.1038/boneres.2016.44 28149655 PMC5240031

[B6] Chou C. H. Jain V. Gibson J. Attarian D. E. Haraden C. A. Yohn C. B. (2020). Synovial cell cross-talk with cartilage plays a major role in the pathogenesis of osteoarthritis. Sci. Rep. 10 (1), 10868. 10.1038/s41598-020-67730-y 32616761 PMC7331607

[B7] Dexheimer V. Gabler J. Bomans K. Sims T. Omlor G. Richter W. (2016). Differential expression of TGF-β superfamily members and role of Smad1/5/9-signalling in chondral versus endochondral chondrocyte differentiation. Sci. Rep. 6 (36655), 36655. 10.1038/srep36655 27848974 PMC5111074

[B8] Fadda S. M. H. Bassyouni I. H. Khalifa R. H. Elsaid N. Y. (2018). Pleiotrophin, the angiogenic and mitogenic growth factor: Levels in serum and synovial fluid in rheumatoid arthritis and osteoarthritis: And correlation with clinical, laboratory and radiological indices. Z Rheumatol. 77 (4), 322–329. 10.1007/s00393-016-0234-8 27904997

[B9] Ferguson C. M. Schwarz E. M. Reynolds P. R. Puzas J. E. Rosier R. N. O'Keefe R. J. (2000). Smad2 and 3 mediate transforming growth factor-beta1-induced inhibition of chondrocyte maturation. Endocrinology 141 (12), 4728–4735. 10.1210/endo.141.12.7848 11108288

[B10] Finnson K. W. Parker W. L. ten Dijke P. Thorikay M. Philip A. (2008). ALK1 opposes ALK5/Smad3 signaling and expression of extracellular matrix components in human chondrocytes. J. Bone Min. Res. 23 (6), 896–906. 10.1359/jbmr.080209 18333754

[B11] Fukada M. Fujikawa A. Chow J. P. H. Ikematsu S. Sakuma S. Noda M. (2006). Protein tyrosine phosphatase receptor type Z is inactivated by ligand-induced oligomerization. FEBS Lett. 580 (17), 4051–4056. 10.1016/j.febslet.2006.06.041 16814777

[B12] Gao S. G. Li K. H. Zeng K. B. Tu M. Xu M. Lei G. H. (2010). Elevated osteopontin level of synovial fluid and articular cartilage is associated with disease severity in knee osteoarthritis patients. Osteoarthr. Cartil. 18 (1), 82–87. 10.1016/j.joca.2009.07.009 19747583

[B13] Huang J. G. Xia C. Zheng X. P. Yi T. T. Wang X. Y. Song G. (2011). 17β-Estradiol promotes cell proliferation in rat osteoarthritis model chondrocytes via PI3K/Akt pathway. Cell Mol. Biol. Lett. 16 (4), 564–575. 10.2478/s11658-011-0023-y 21847664 PMC6275964

[B14] Ji Q. Zheng Y. Zhang G. Hu Y. Fan X. Hou Y. (2019). Single-cell RNA-seq analysis reveals the progression of human osteoarthritis. Ann. Rheum. Dis. 78 (1), 100–110. 10.1136/annrheumdis-2017-212863 30026257 PMC6317448

[B15] Jin S. Guerrero-Juarez C. F. Zhang L. Chang I. Ramos R. Kuan C. H. (2021). Inference and analysis of cell-cell communication using CellChat. Nat. Commun. 12 (1), 1088–21246. 10.1038/s41467-021-21246-9 33597522 PMC7889871

[B16] Kaspiris A. Mikelis C. Heroult M. Khaldi L. Grivas T. B. Kouvaras I. (2013). Expression of the growth factor pleiotrophin and its receptor protein tyrosine phosphatase beta/zeta in the serum, cartilage and subchondral bone of patients with osteoarthritis. Jt. Bone Spine 80 (4), 407–413. 10.1016/j.jbspin.2012.10.024 23333521

[B17] Kim M. K. Ha C. W. Cho S. D. Choi E. S. Ha J. K. (2018). A multicenter, double-blind, phase III clinical trial to evaluate the efficacy and safety of a cell and gene therapy in knee osteoarthritis patients. Hum. Gene Ther. Clin. Dev. 29 (1), 48–59. 10.1089/humc.2017.249 29641281

[B18] Kim S. Kang D. Huo Z. Park Y. Tseng G. C. (2018). Meta-analytic principal component analysis in integrative omics application. Bioinformatics 34 (8), 1321–1328. 10.1093/bioinformatics/btx765 29186328 PMC5905607

[B19] Korsunsky I. Millard N. Fan J. Slowikowski K. Zhang F. Wei K. (2019). Fast, sensitive and accurate integration of single-cell data with Harmony. Nat. Methods 16 (12), 1289–1296. 10.1038/s41592-019-0619-0 31740819 PMC6884693

[B20] Laiguillon M. C. Houard X. Bougault C. Gosset M. Nourissat G. Sautet A. (2014). Expression and function of visfatin (Nampt), an adipokine-enzyme involved in inflammatory pathways of osteoarthritis. Arthritis Res. Ther. 16 (1), R38. 10.1186/ar4467 24479481 PMC3978827

[B21] Li T. F. Darowish M. Zuscik M. J. Chen D. Schwarz E. M. Rosier R. N. (2006). Smad3-deficient chondrocytes have enhanced BMP signaling and accelerated differentiation. J. Bone Min. Res. 21 (1), 4–16. 10.1359/JBMR.050911 PMC2649698 16355269

[B22] Li X. Liao Z. Deng Z. Chen N. Zhao L. (2021). Combining bulk and single-cell RNA-sequencing data to reveal gene expression pattern of chondrocytes in the osteoarthritic knee. Bioengineered 12 (1), 997–1007. 10.1080/21655979.2021.1903207 33749514 PMC8806218

[B23] Li Y. S. Xiao W. Sun M. Deng Z. Zeng C. Li H. (2016). The expression of osteopontin and Wnt5a in articular cartilage of patients with knee osteoarthritis and its correlation with disease severity. Biomed. Res. Int. 2016, 9561058. 10.1155/2016/9561058 27556044 PMC4983346

[B24] Liang J. Xu L. Zhou F. Liu A. M. Ge H. X. Chen Y. Y. (2018). MALAT1/miR-127-5p regulates osteopontin (OPN)-Mediated proliferation of human chondrocytes through PI3K/akt pathway. J. Cell Biochem. 119, 431–439. 10.1002/jcb.26200 28590075

[B25] Liao L. Chen Y. Wang W. (2016). The current progress in understanding the molecular functions and mechanisms of visfatin in osteoarthritis. J. Bone Min. Metab. 34 (5), 485–490. 10.1007/s00774-016-0743-1 26969394

[B26] Liedert A. Schinke T. Ignatius A. Amling M. (2014). The role of midkine in skeletal remodelling. Br. J. Pharmacol. 171 (4), 870–878. 10.1111/bph.12412 24102259 PMC3925025

[B27] Lin C. Chen Z. Guo D. Zhou L. Lin S. Li C. (2022). Increased expression of osteopontin in subchondral bone promotes bone turnover and remodeling, and accelerates the progression of OA in a mouse model. Aging 14 (1), 253–271. 10.18632/aging.203707 34982732 PMC8791213

[B28] Lin C. Shao Y. Zeng C. Zhao C. Fang H. Wang L. (2018). Blocking PI3K/AKT signaling inhibits bone sclerosis in subchondral bone and attenuates post-traumatic osteoarthritis. J. Cell Physiol. 233 (8), 6135–6147. 10.1002/jcp.26460 29323710

[B29] Liu Q. Zeng H. Yuan Y. Wang Z. Wu Z. Luo W. (2020). Osteopontin inhibits osteoarthritis progression via the OPN/CD44/PI3K signal axis. Genes Dis. 9 (1), 128–139. 10.1016/j.gendis.2020.06.006 35005113 PMC8720673

[B30] Long H. Liu Q. Yin H. Wang K. Diao N. Zhang Y. (2022). Prevalence trends of site-specific osteoarthritis from 1990 to 2019: Findings from the global burden of disease study 2019. Arthritis Rheumatol. 74 (7), 1172–1183. 10.1002/art.42089 35233975 PMC9543105

[B31] Lu D. R. Wu H. Driver I. Ingersoll S. Sohn S. Wang S. (2020). Dynamic changes in the regulatory T-cell heterogeneity and function by murine IL-2 mutein. Life Sci. Alliance 3 (5), e201900520. 10.26508/lsa.201900520 32269069 PMC7156283

[B32] MacDonald I. J. Liu S. C. Su C. M. Wang Y. H. Tsai C. H. Tang C. H. (2018). Implications of angiogenesis involvement in arthritis. Int. J. Mol. Sci. 19 (7), 2012. 10.3390/ijms19072012 29996499 PMC6073145

[B33] Min S. Shi T. Han X. Chen D. Xu Z. Shi D. (2021). Serum levels of leptin, osteopontin, and sclerostin in patients with and without knee osteoarthritis. Clin. Rheumatol. 40 (1), 287–294. 10.1007/s10067-020-05150-z 32588275

[B34] Mukherjee S. Heath L. Preuss C. Jayadev S. Garden G. A. Greenwood A. K. (2020). Molecular estimation of neurodegeneration pseudotime in older brains. Nat. Commun. 11 (1), 5781–19622. 10.1038/s41467-020-19622-y 33188183 PMC7666177

[B35] Nanus D. E. Badoume A. Wijesinghe S. N. Halsey A. M. Hurley P. Ahmed Z. (2021). Synovial tissue from sites of joint pain in knee osteoarthritis patients exhibits a differential phenotype with distinct fibroblast subsets. EBioMedicine 72, 103618. 10.1016/j.ebiom.2021.103618 34628351 PMC8511845

[B36] Peterson W. J. Tachiki K. H. Yamaguchi D. T. (2004). Serial passage of MC3T3-E1 cells down-regulates proliferation during osteogenesis *in vitro* . Cell Prolif. 37 (5), 325–336. 10.1111/j.1365-2184.2004.00316.x 15377332 PMC6495877

[B37] Philp A. M. Butterworth S. Davis E. T. Jones S. W. (2021). eNAMPT is localised to areas of cartilage damage in patients with hip osteoarthritis and promotes cartilage catabolism and inflammation. Int. J. Mol. Sci. 22 (13), 6719. 10.3390/ijms22136719 34201564 PMC8269388

[B38] Pufe T. Bartscher M. Petersen W. Tillmann B. Mentlein R. (2003). Pleiotrophin, an embryonic differentiation and growth factor, is expressed in osteoarthritis. Osteoarthr. Cartil. 11 (4), 260–264. 10.1016/s1063-4584(02)00385-0 12681952

[B39] Pufe T. Groth G. Goldring M. B. Tillmann B. Mentlein R. (2007). Effects of pleiotrophin, a heparin-binding growth factor, on human primary and immortalized chondrocytes. Osteoarthr. Cartil. 15 (2), 155–162. 10.1016/j.joca.2006.07.005 16949312

[B40] Qiu X. Mao Q. Tang Y. Wang L. Chawla R. Pliner H. A. (2017). Reversed graph embedding resolves complex single-cell trajectories. Nat. Methods 14 (10), 979–982. 10.1038/nmeth.4402 28825705 PMC5764547

[B41] Rannou F. Pelletier J. P. Martel-Pelletier J. (2016). Efficacy and safety of topical NSAIDs in the management of osteoarthritis: Evidence from real-life setting trials and surveys. Semin. Arthritis Rheum. 45 (4), S18–S21. 10.1016/j.semarthrit.2015.11.007 26806189

[B42] Robbins Y. Friedman J. Clavijo P. E. Sievers C. Bai K. Donahue R. N. (2021). Dual PD-L1 and TGF-b blockade in patients with recurrent respiratory papillomatosis. J. Immunother. Cancer 9 (8), e003113. 10.1136/jitc-2021-003113 34462327 PMC8407210

[B43] Ruan M. Z. Erez A. Guse K. Dawson B. Bertin T. Chen Y. (2013). Proteoglycan 4 expression protects against the development of osteoarthritis. Sci. Transl. Med. 5 (176), 176ra34. 10.1126/scitranslmed.3005409 PMC3804124 23486780

[B44] Satija R. Farrell J. A. Gennert D. Schier A. F. Regev A. (2015). Spatial reconstruction of single-cell gene expression data. Nat. Biotechnol. 33 (5), 495–502. 10.1038/nbt.3192 25867923 PMC4430369

[B45] Sebastian A. McCool J. L. Hum N. R. Murugesh D. K. Wilson S. P. Christiansen B. A. (2021). Single-cell RNA-seq reveals transcriptomic heterogeneity and post-traumatic osteoarthritis-associated early molecular changes in mouse articular chondrocytes. Cells 10 (6), 1462. 10.3390/cells10061462 34200880 PMC8230441

[B46] Sheng X. Lin Z. Lv C. Shao C. Bi X. Deng M. (2020). Cycling stem cells are radioresistant and regenerate the intestine. Cell Rep. 32 (4), 107952. 10.1016/j.celrep.2020.107952 32726617 PMC7789978

[B47] Sun K. Luo J. Guo J. Yao X. Jing X. Guo F. (2020). The PI3K/AKT/mTOR signaling pathway in osteoarthritis: A narrative review. Osteoarthr. Cartil. 28 (4), 400–409. 10.1016/j.joca.2020.02.027 32081707

[B48] Thielen N. G. M. Neefjes M. Vitters E. L. van Beuningen H. M. Blom A. B. Koenders M. I. (2022). Identification of transcription factors responsible for a transforming growth factor-β-driven hypertrophy-like phenotype in human osteoarthritic chondrocytes. Cells 11 (7), 1232. 10.3390/cells11071232 35406794 PMC8998018

[B49] Tian J. Cheng C. Kuang S. D. Su C. Zhao X. Xiong Y. L. (2020). OPN deficiency increases the severity of osteoarthritis associated with aberrant chondrocyte senescence and apoptosis and upregulates the expression of osteoarthritis-associated genes. Pain Res. Manag. 22 (3428587), 3428587. 10.1155/2020/3428587 PMC7599390 33144900

[B50] Tominaga K. Suzuki H. I. (2019). TGF-Β signaling in cellular senescence and aging-related Pathology. Int. J. Mol. Sci. 20 (20), 5002. 10.3390/ijms20205002 31658594 PMC6834140

[B51] van der Kraan P. M. (2017). The changing role of TGFβ in healthy, ageing and osteoarthritic joints. Nat. Rev. Rheumatol. 13 (3), 155–163. 10.1038/nrrheum.2016.219 28148919

[B52] Wang X. (2020). Pleiotrophin: Activity and mechanism. Adv. Clin. Chem. 98, 51–89. 10.1016/bs.acc.2020.02.003 32564788 PMC7672882

[B53] Woetzel D. Huber R. Kupfer P. Pohlers D. Pfaff M. Driesch D. (2014). Identification of rheumatoid arthritis and osteoarthritis patients by transcriptome-based rule set generation. Arthritis Res. Ther. 16 (2), R84. 10.1186/ar4526 24690414 PMC4060460

[B54] Yang S. Ryu J. H. Oh H. Jeon J. Kwak J. S. Kim J. H. (2015). NAMPT (visfatin), a direct target of hypoxia-inducible factor-2α, is an essential catabolic regulator of osteoarthritis. Ann. Rheum. Dis. 74 (3), 595–602. 10.1136/annrheumdis-2013-204355 24347567 PMC4345811

[B55] Yao X. Zhang J. Jing X. Ye Y. Guo J. Sun K. (2019). Fibroblast growth factor 18 exerts anti-osteoarthritic effects through PI3K-AKT signaling and mitochondrial fusion and fission. Pharmacol. Res. 139, 314–324. 10.1016/j.phrs.2018.09.026 30273654

[B56] Yumoto K. Ishijima M. Rittling S. R. Tsuji K. Tsuchiya Y. Kon S. (2002). Osteopontin deficiency protects joints against destruction in anti-type II collagen antibody-induced arthritis in mice. Proc. Natl. Acad. Sci. U. S. A. 99 (7), 4556–4561. 10.1073/pnas.052523599 11930008 PMC123686

