# Supplementary material for: Single-cell RNA sequencing analysis of human chondrocytes reveals cell–cell communication alterations mediated by interactive signaling pathways in osteoarthritis
Source: Front Cell Dev Biol. 2023 Apr 4;11:1099287. doi: 10.3389/fcell.2023.1099287 (PMC10112522; doi:10.3389/fcell.2023.1099287)
Supplement: Supplementary file 1 [file DataSheet3.docx]

Supplementary Material

**
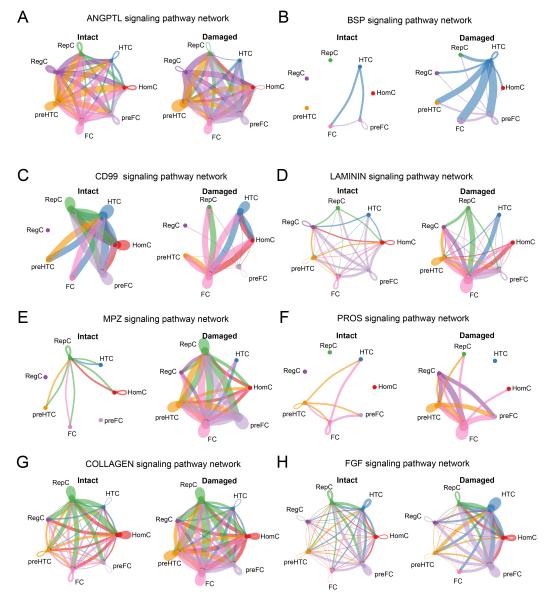
**

#### **Supplementary Figure 1.** Cellular communication changes among the chondrocyte subtypes mediated by multiple signaling pathways. Colored dots represent the different cell subtypes. ANGPTL:angiopoietin like, BSP:black spleen, MPZ:[myelin protein zero](https://www.ncbi.nlm.nih.gov/gene/4359), PROS:proline--tRNA ligase ,FGF:fibroblast growth factor.

**
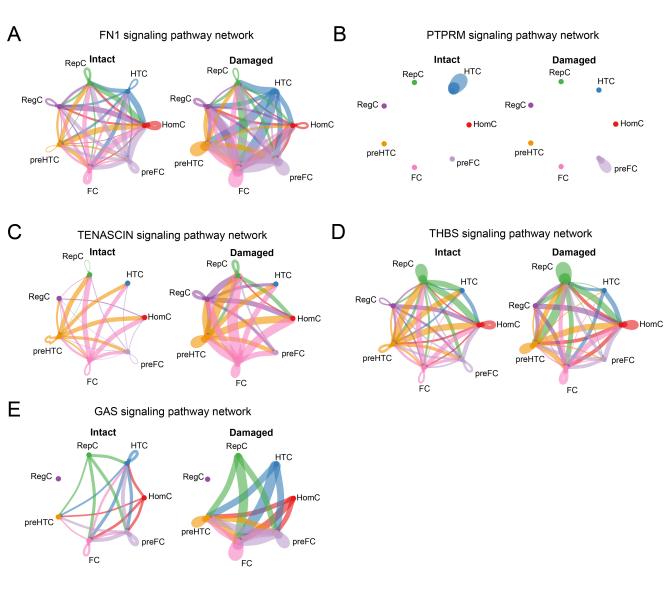
**

**Supplementary** **Figure 2.** Cellular communication changes among the chondrocyte subtypes mediated by multiple signaling pathways. Colored dots represent the different cell subtypes. FN1:[fibronectin 1](https://www.ncbi.nlm.nih.gov/gene/2335), PTPRM:[protein tyrosine phosphatase receptor type M](https://www.ncbi.nlm.nih.gov/gene/5797), THBS:thrombospondin, GAS:gastrin.
