# Supplementary figures and images for: Single-cell RNA sequencing analysis of human chondrocytes reveals cell–cell communication alterations mediated by interactive signaling pathways in osteoarthritis
Source: Front Cell Dev Biol. 2023 Apr 4;11:1099287. doi: 10.3389/fcell.2023.1099287 (PMC10112522; doi:10.3389/fcell.2023.1099287)

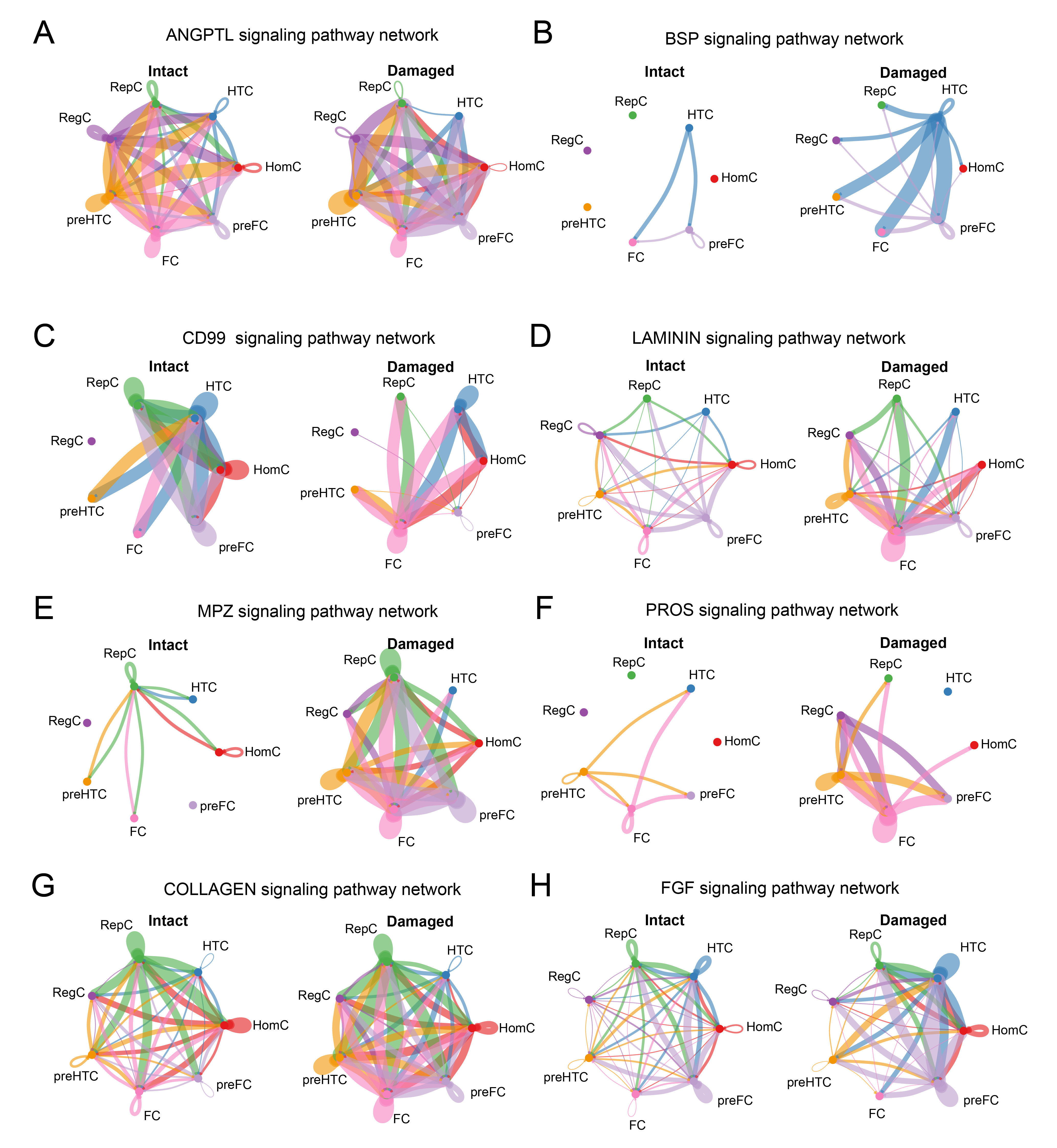

Supplement: Supplementary file 2 [file Image1.JPEG]

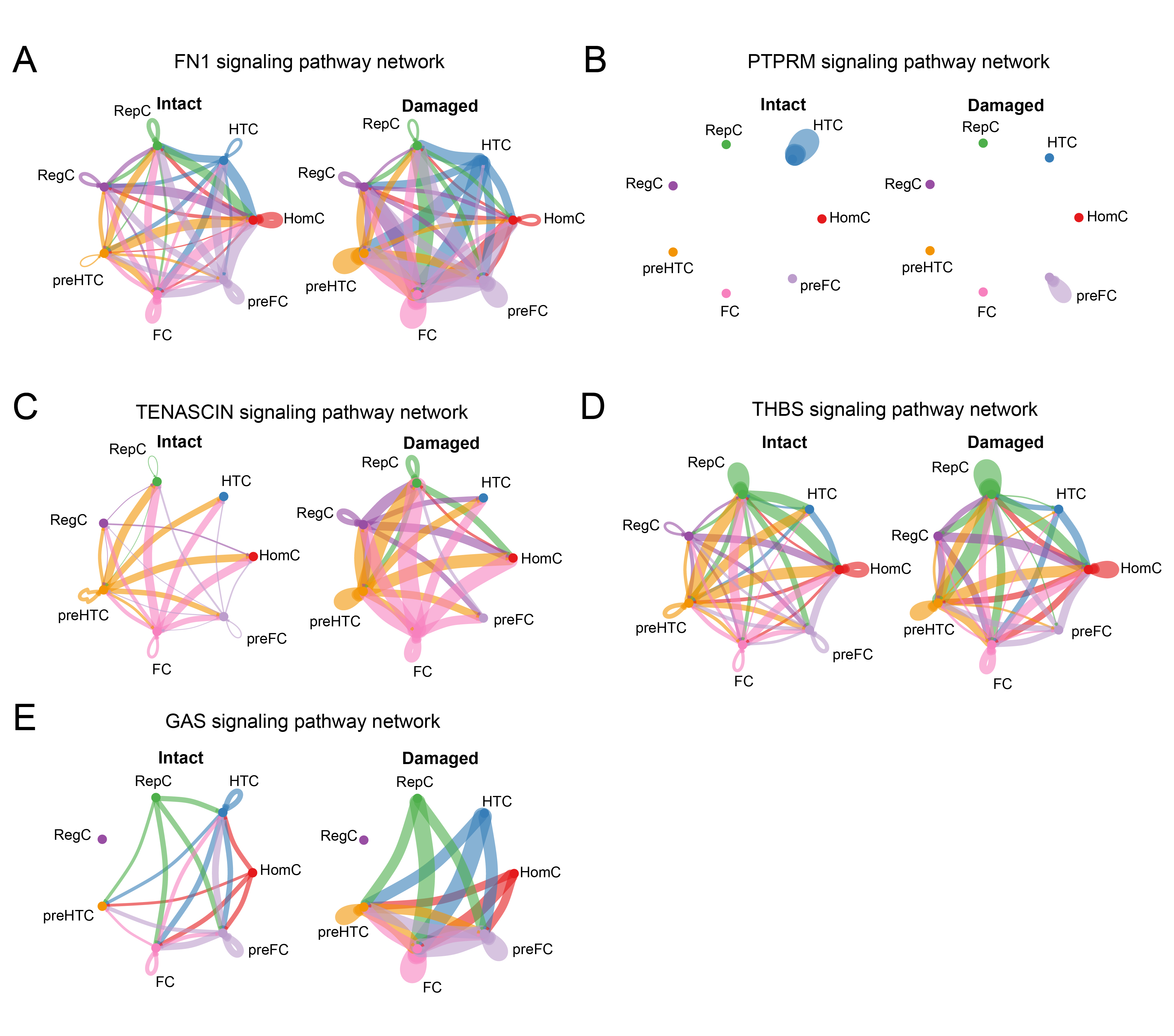

Supplement: Supplementary file 3 [file Image2.JPEG]
