# Supplementary material for: Single-cell RNA sequencing analysis of human chondrocytes reveals cell–cell communication alterations mediated by interactive signaling pathways in osteoarthritis
Source: Front Cell Dev Biol. 2023 Apr 4;11:1099287. doi: 10.3389/fcell.2023.1099287 (PMC10112522; doi:10.3389/fcell.2023.1099287)
Supplement: Supplementary file 4 [file DataSheet2.docx]

Supplementary Material 2

The tSNE plot of cell type distribution in Damaged and Intact. The tSNE analysis between damaged and control is shown in the figure below, showed no significant difference in cell distribution, but the cell number of HomC, HTC, and RegC was reduced in damaged.


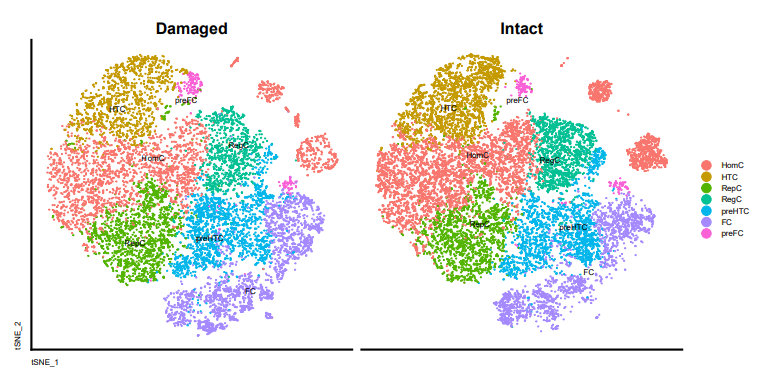


Number of 7 cell types in Damaged and Intact:

|  | HomC | HTC | RepC | RegC | preHTC | FC | preFC |
| --- | --- | --- | --- | --- | --- | --- | --- |
| Damaged | 2684 | 1306 | 1617 | 983 | 1851 | 2250 | 236 |
| Intact | 4975 | 2152 | 1725 | 1461 | 1868 | 1934 | 203 |
