# Supplementary material for: Single-cell RNA sequencing analysis of human chondrocytes reveals cell–cell communication alterations mediated by interactive signaling pathways in osteoarthritis
Source: Front Cell Dev Biol. 2023 Apr 4;11:1099287. doi: 10.3389/fcell.2023.1099287 (PMC10112522; doi:10.3389/fcell.2023.1099287)
Supplement: Supplementary file 5 [file DataSheet1.docx]

Supplementary Material 1

Single-cell dataset GSE152805 source:“Matched articular cartilage, synovial membrane and synovial fluid biospecimens were acquired as anonymized surgical waste from 22 patients, mean age 69.1 (SD 7.0) years, 72% (n = 16) female, mean BMI 29.3 (SD 5.9) kg/m2, all undergoing total knee replacement for medial compartment dominant knee OA. Joint tissues (cartilage and synovial membrane) from 3 OA patients of mean age 67.7 (SD 2.31) years, 2 female, BMI 39 (SD 4.6) kg/m2 were randomly selected, from the total of 22, for scRNA-seq analysis.” Only the chondrocytes were used in our analysis.

General transcriptomic dataset GSE55235 Source: “Synovial membrane samples were obtained either from postmortem joints/traumatic joint injury cases (control group (CG); n = 15 and n = 5, respectively) or from RA/OA patients (all Caucasian) upon joint replacement/synovectomy at the Jena University Hospital, Chair of Orthopedics, Waldkrankenhaus ‘Rudolf Elle’, Eisenberg, Germany (n = 33, dataset ‘Jena’), at the Department of Orthopedics/Institute of Pathology/Department of Rheumatology and Clinical Immunology, Charité-Universitätsmedizin Berlin (n = 30, dataset ‘Berlin’), and at the Department of Orthopedics/ Institute of Pathology, University of Leipzig (n = 16, dataset ‘Leipzig’).” We analyzed an analysis of 10 normal samples and 10 osteoarthritis samples.
